# An intentional cohesion call in male chimpanzees of Budongo Forest

**DOI:** 10.1007/s10071-022-01597-6

**Published:** 2022-01-19

**Authors:** Alice Bouchard, Klaus Zuberbühler

**Affiliations:** 1 Institute of Biology, University of Neuchatel, Neuchâtel, Switzerland; 2 School of Psychology and Neuroscience, University of St Andrews, St Andrews, Scotland, UK; 3 Budongo Conservation Field Station, Masindi, Uganda

**Keywords:** *Pan troglodytes*, Intentionality, Vocalisation, Social cognition, Group cohesion

## Abstract

Many social animals travel in cohesive groups but some species, including chimpanzees, form flexible fission–fusion systems where individuals have some control over group cohesion and proximity to others. Here, we explored how male chimpanzees of the Sonso community of Budongo Forest, Uganda, use communication signals during resting, a context where the likelihood of group fission is high due to forthcoming travel. We focused on a context-specific vocalisation, the ‘rest hoo’, to investigate its function and determine whether it is produced intentionally. We found that this call was typically given towards the end of typical silent resting bouts, i.e., the period when individuals need to decide whether to continue travelling after a brief stop-over or to start a prolonged resting bout. Subjects rested longer after producing ‘rest hoos’ and their resting time increased with the number of calls produced. They also rested longer if their calls were answered. Furthermore, focal subjects’ resting time was prolonged after hearing others’ ‘rest hoos’. Subjects called more when with top proximity partners and in small parties and rested longer if a top proximity partner called. We also found an interaction effect between rank and grooming activity, with high-ranking males with a high grooming index calling less frequently than other males, suggesting that vocal communication may serve as a cohesion strategy alternative to tactile-based bonding. We discuss these different patterns and conclude that chimpanzee ‘rest hoos’ meet key criteria for intentional signalling. We suggest that ‘rest hoos’ are produced to prolong resting bouts with desired partners, which may function to increase social cohesion.

**Supplementary Information:**

The online version contains supplementary material available at 10.1007/s10071-022-01597-6.

## Introduction

A major challenge for group-living animals is to balance the social need for cohesion with sometimes diverging individual needs of resting, feeding or moving (Kerth 2010; Petit and Bon 2010). Fission–fusion societies are an evolutionary response to these opposing demands, enabling group members to take individual decisions depending on local food availability (Asensio et al. 2009; Chapman et al. 1995; Kummer 1971; Sueur et al. 2011; Symington 1990) and social needs, as documented in various species (bottlenose dolphins: Lusseau 2007; spider monkeys: Busia et al. 2017; chimpanzees: Mitani and Amsler 2003). In these social systems, decision-making is devolved from the collective to the private, from a small number of influential decision-makers to most group members (Conradt and List 2009; Couzin et al. 2005; Stueckle and Zinner 2008). Compared to the more standard social system, i.e., cohesive social groups, fission–fusion systems are more likely to lead to disagreement, negotiation and coordination (Kerth 2010), and this may put higher demands on social cognition and socially targeted communication (Aureli et al. 2008; Couzin et al. 2005; Strandburg-Peshkin et al. 2018). Correspondingly, and in line with the social intelligence hypothesis (Humphrey 1976), species with high degrees of fission–fusion dynamics, such as spider monkeys (Aguilar-Melo et al. 2018), baboons (Kummer 1968), bonobos (White and Burgman 1990), chimpanzees (Goodall 1986), dolphins (Tsai and Mann 2013) or humans (Marlowe 2005), all have relatively large neocortices if compared to species living in more cohesive groups (Barrett et al. 2003; Dunbar 1998).

How do individuals adjust social cohesion? Many group-living species have evolved acoustically distinct vocalisations that function for this purpose, typically referred to as ‘contact calls’ (Bolt 2020; Chaverri et al. 2013; da Cunha and Byrne 2009). These calls usually provide information about the identity of the caller and, in some instances, about the caller’s current activity (Candiotti et al. 2012; Fischer 2008) or desired response (Gruber and Zuberbühler 2013). In fission–fusion societies, however, patterns of cohesion are more complex and may thus require more complex communication. Chimpanzees, for instance, maintain contact with distant group members with acoustically distinct long-distance calls (i.e., ‘pant-hoots’) and call exchanges are more frequent between preferred social partners than other group members (Eckhardt et al. 2015; Mitani and Nishida 1993). During close-range interactions, chimpanzees use a range of other functionally distinct vocalisations, such as ‘travel hoos’, to coordinate departures or ‘rough grunts’ during feeding. These calls seem to be produced in goal-directed ways, such as to initiate travel or coordinate feeding, and are preferentially given in the presence of preferred social partners, suggesting that they possess some of the hallmarks of intentionality (Bates 1979; Fedurek and Slocombe 2013; Gruber and Zuberbühler 2013; Schel et al. 2013a).

In previous studies, it has been noted that, when resting, chimpanzees often produce a distinct vocalisation, the ‘rest hoo’ (Crockford et al. 2015; Gruber and Zuberbühler 2013). In this study, we were interested in investigating the function of ‘rest hoo’ vocalisations and determining whether such communication behaviour qualifies as intentional. Intentionality in animal communication usually refers to signals produced voluntarily by a signaller in an attempt to manipulate the recipient’s behaviour (Bard 1990; Tomasello et al. 1985). While intentionality has been of interest to philosophy, empirically the concept is best investigated during natural communication acts. Here, pioneering work by Bates (1979) has proposed a definition based on key behavioural markers, i.e., a signal must be goal-directed and produced in the presence of an audience to qualify both as intentional and communicative (see Leavens et al. 2005; Leavens and Hopkins 1998). More recently, other elements of definitions have been proposed, such as the requirement that the recipient responds in line with the signaller’s presumed goal, a contentious issue because recipients may be in conflict with the signaller’s intention and, therefore, choose not to respond (see Liebal et al. 2014; Townsend et al. 2017).

The aim of this study was to investigate the function of ‘rest hoo’ vocalisations and whether they qualified as intentional signals. To do so, we observed a group of wild chimpanzees in Budongo Forest (Uganda) for over a year, where the demography and social relations between the individuals are known. Chimpanzees travel over considerable distances to visit food trees, interspersed by sometimes long resting periods, which account for the largest proportion of their day-time activity budgets (about 45% of time, A Bouchard, unpublished data; see also Kosheleff and Anderson 2009; Yamanashi and Hayashi 2011). Changes in cohesion and group composition are most common during activity changes, such as when transitioning between travelling and resting.

Chimpanzees can produce acoustically distinct vocalisations in these situations, notably ‘rest hoos’ during resting and ‘travel hoos’ before or during travel. A third call type that is acoustically similar is given in response to mild dangers (i.e., ‘alert hoo’). All three ‘hoo’ variants are close-range calls (< 150 m; Crockford et al. 2015, 2018) and are acoustically distinguishable, with significant variations in call durations, maximum fundamental frequencies and inter-call intervals (Crockford et al. 2018). Previous studies have suggested that ‘travel’ and ‘alert hoos’ are goal-directed and produced in the presence of an audience, suggesting that they qualify as intentional signals (sensu Bates 1979). Even though ‘rest hoos’ have been previously mentioned in the literature, sometimes with a different name (e.g., ‘extended grunt’; Goodall 1986; Laporte 2010; Mullins 2014), their function had never been investigated.

We focused on adult males due to the fact that they form stronger social bonds with each other than with females (Gilby and Wrangham 2008; Mitani 2009; Wrangham et al. 1992). Bonding in male chimpanzees is important for a variety of activities and serves as a basis for trust and support during dangerous activities, such as intergroup aggression (Herbinger et al. 2009; Wilson et al. 2014), cooperative hunting (Hobaiter et al. 2017), predator defence (Boesch 1991) and intragroup conflicts (Goodall 1986; Mitani 2009; Muller and Mitani 2005). Generally, associating with high-ranking males can also be advantageous and bring fitness benefits, such as higher reproductive success (Bray et al. 2016; Duffy et al. 2007; Feldblum et al. 2021; Kaburu and Newton-Fisher 2015).

For the aforementioned reasons, we thus expected males to care about maintaining proximity to desirable individuals, i.e., preferred social partners and high-ranking males. Since group fissions are likely to happen before and after resting bouts, males are likely to be especially challenged in these situations. We tested the hypothesis that chimpanzees produced ‘rest hoos’ to communicate their intention to keep resting with specific individuals (i.e., that calls are goal-directed). In relation to this, we predicted that subjects should prolong resting bouts after producing ‘rest hoos’ and that these calls should be produced in the presence of desirable audiences, i.e., preferred social partners and high-ranking males. To test these predictions, we analysed how call production affected a signaller’s resting time in different audiences. We also explored individual variation in call production, focusing on individuals’ rank and grooming index. Finally, we predicted that, if ‘rest hoos’ were produced intentionally, the behavioural response of recipients should mainly be in line with the signaller’s goal, i.e., recipients should on average prolong their resting time. We thus analysed the recipients resting time in relation to ‘rest hoo’ production and the signaller’s identity.

## Methods

### Study site and subjects

Data were collected between January 2018 and March 2020 on East African chimpanzees (*Pan troglodytes schweinfurthii*) of the Sonso community in the Budongo Forest Reserve, Uganda (latitude 1°37′–2°00′ N; longitude: 31°22′–31°46′ E). The community has a home range of around 7 km^2^ (Newton-Fisher 2003) and has been well habituated to human presence for more than 25 years. All individuals were identified and most of their social and kin relations were known (Reynolds 2005). At the beginning of the study, the community consisted of 75 individuals, including 37 adults (> 15 years, 11 males). The study subjects were the 11 adult males (although 3 of them died in an epidemic in February 2019).

### Data collection

We conducted full-day (usually between 07:00 and 16:30 local time) focal follows of 11 adult male chimpanzees. One male (ZD) was excluded from analysis due to low observation time (Table 1). During each follow, we continuously recorded the subject’s activities (feed, travel, and rest) with start and end times. For each resting bout (defined as the subject stopping for at least 15 s), we recorded the duration, initial party composition and time of ‘rest hoo’ production by the subject and by any other individual throughout the bout. For each ‘rest hoo’, we also recorded the identity of the caller and whether it elicited an immediate vocal response within the same vocal bout (i.e., within the next 5 s). We excluded resting bouts during which ‘rest hoos’ were produced by unidentifiable individuals. We also recorded if there was a change of activity (i.e., rest, travel or feed) in the audience immediately (i.e., within 5 s) before or after call production. Audio recordings of ‘rest hoo’ vocalisations are included in the electronic supplementary material.

**Table 1:** Adult male chimpanzees that participated in the study, with their age (at the beginning of the study), the total focal time, the number of ‘rest hoo’ calls recorded (during the focal data collection), their elo-rating scores and dominance, as well as their grooming index

Focal ID	Age	Focal time (h)	Number of ‘rest hoos’ produced	Elo-rating	Dominance	Grooming index
HW	24	88	15	1758	High-ranking	1.32897
FK	18	60	37	1361	High-ranking	− 0.43526
MS	26	87	35	1316	High-ranking	1.25555
SM	24	63	26	1028	Non-dominant	− 1.11299
SQ	26	55	22	962	Non-dominant	0.68209
ZL	21	66	22	958	Non-dominant	0.53392
PS	19	87	27	915	Non-dominant	− 0.34498
KT	23	83	25	893	Non-dominant	− 1.00136
ZF	35	22	18	834	Non-dominant	− 0.3755
KZ	22	71	41	493	Non-dominant	0.97484
ZD^a^	16	10	0	482	Non-dominant	− 1.50527

^a^Excluded from analysis due to low focal time

### Social variables

To establish the social relations between individuals, we used long-term data collected by four trained field assistants to determine each subject’s dominance rank (Elo-rating), grooming index and preferred social partners (grooming and proximity partners). Similarly to Samuni et al. (2018, 2021), we did not calculate an overall sociality index but two separate indices; one based on grooming interactions (i.e., grooming partners) and another based on spatial proximity (i.e., proximity partners). Several studies have shown that relationships formed through mere spatial proximity can be different from those involving tactile grooming and, therefore, may affect individual behaviours differently (Bray and Gilby 2020; Mitani 2009; Samuni et al. 2018, 2021), including vocal behaviour (Mitani and Nishida 1993).

Data collection was during full-day focal follows to document all affiliative and agonistic interactions with every other individual in the group. Party composition (i.e., all individuals within 30 m of the focal) was continuously monitored and determined every 15 min during scan sampling. Due to a respiratory disease outbreak in 2019, three of the focal animals died, which destabilised the hierarchy and social relations between the remaining males. For this reason, we could only establish social relationships among males for the pre-outbreak period, which reduced the dataset for some analyses. Specifically, when investigating the effects of social relationships on call production or reception, we only analysed data collected until 26 February 2019 (GLM1, GLMM6, GLMM8, see below).

#### Dominance

The hierarchy of the different adult males was determined using the Elo-rating method (Elo 1978; Neumann et al. 2011) on ‘pant grunt’ data (i.e., a ‘greeting’ vocalisation given to higher-ranking individuals), a method that has been validated against other commonly used methods (Neumann et al. 2011). To have an accurate estimation of the dominance ranks at the beginning of the study, we used data from the 12 months before the beginning of the study followed by data collected until the end of the study period (January 4th 2017 to February 26th 2019). We calculated the Elo-ratings using the ‘EloRating’ R package version 0.46.11 (Neumann and Kulik 2020). The hierarchy was stable through the 2017–2019 study period, enabling us to attribute a final Elo-score to each male (i.e., the Elo-score obtained at the end of the observation period). Three males had consistently higher Elo-scores than the other males and were thus classified as ‘high-ranking’ (Table 1; Online Resource 1, Fig. S1). Elo-rating scores were standardized for subsequent statistical analyses.

#### Grooming index

We determined each adult male’s grooming index, which indicated how much time he spent grooming relative to the other adult males of the group. We were interested in grooming interactions, rather than the directionality of grooming, so both grooming given and received (with any individual in the group) were considered. The grooming index was calculated from the focal data as a corrected standardized grooming rate (grooming duration per observation time) using the ‘socialindices2’ R package version 0.50.0 (Neumann 2017) (Table 1).

#### Grooming and proximity partners

To determine the preferred grooming and proximity partners between all male dyads, we calculated a grooming-based and a proximity-based dyadic sociality indices for each male dyad, the DSI_G_ and the DSI_P_ (based on DSI; see Silk et al. 2013 and Online Resource 1, Table S1). To calculate the DSI_G_, we used the grooming data, defined as the duration of grooming interactions (grooming given or received) between the two males of the dyad, recorded during the focal follows. To calculate the DSI_P_, we used the nearest neighbour data, defined as the individual sitting in closest proximity to the focal animal during each 15 min scan, considering only data where the nearest neighbour was another adult male. The DSI_G_ and the DSI_P_ were calculated using the ‘socialindices2’ R package version 0.50.0 (Neumann 2017). For each focal male, the top three grooming partners and the top three proximity partners were the top three individuals who had the highest DSI_G_ and DSI_P_ values, respectively (Online Resource 1, Table S1).

### Statistical analyses

#### ‘Rest hoo’ production

To establish if the subjects produced ‘rest hoos’ in the presence of an audience, we analysed all resting bouts (i.e., with or without ‘rest hoo’ production) and ran a generalised linear mixed model (GLMM1) with a binomial error structure and logit link function, with the production of ‘rest hoo’ vocalisation (at least one ‘rest hoo’ produced/no ‘rest hoo’ produced) as the response variable. We used the presence of a potential audience (yes/no) as the test variable and the subject ID as a random factor.

To explore which social (i.e., the presence of specific individuals) and individual (i.e., rank and grooming index) parameters influenced whether the subjects produced a ‘rest hoo’ or not, we only analysed resting bouts with a potential audience and excluded events with ‘rest hoos’ produced by other individuals. We ran a generalized linear model (GLM1) with a binomial error structure and logit link function, with the production of ‘rest hoo’ vocalisation (at least one ‘rest hoo’ produced/no ‘rest hoo’ produced) as the response variable. Test variables were the total number of individuals present, the presence of females (yes/no), the presence of a high-ranking male (yes/no), the presence of one of the subject’s top three grooming partners (yes/no), the presence of one of the subject’s top three proximity partners (yes/no), as well as the interaction between the grooming index and the rank of the subject (Elo-rating). The time spent resting (in minutes) was entered as a control variable.

Since we were particularly interested to know if the presence of specific males could affect ‘rest hoo’ production by the subject, we analysed events when the recipient was unambiguous (i.e., when the subject was resting only with one other male). We ran a GLMM (GLMM2) with a binomial error structure and logit link function, with the production of ‘rest hoo’ vocalisation (at least one ‘rest hoo’ produced/no ‘rest hoo’ produced) as the response variable. Whether the resting partner was a high-ranking male (yes/no), one of the subject’s top three grooming partners (yes/no), or top three proximity partners (yes/no) were the test variables. The time spent resting (in minutes) was the control variable and the subject ID was entered as a random factor.

If ‘rest hoos’ were produced to keep spatial proximity with specific partners, we would also expect these partners to keep resting with the subject until the end of the resting bout (i.e., until the subject departs). We thus further analysed these dyadic resting bouts between males and ran a GLMM (GLMM3) with a binomial error structure and logit link function, with the presence of the resting partner at the end of the resting bout (yes/no) as the response variable. Whether the subject produced a ‘rest hoo’ (at least one ‘rest hoo’ produced/no ‘rest hoo’ produced) was the test variable, the time spent resting (in minutes) was the control variable and the subject ID was entered as a random factor.

We then investigated whether subjects prolonged their resting time after producing a ‘rest hoo’ and whether immediate vocal response affected this resting time. If ‘rest hoos’ were produced randomly, the probability of this call being produced would increase with the duration of the resting bouts. Therefore, to avoid this bias, we compared the total duration of resting bouts without ‘rest hoos’ to the duration of resting bouts after ‘rest hoo’ production. To do so, we ran two other GLMMs with a gamma distribution and inverse link function, with the time spent resting (in minutes) after the subject produced his first ‘rest hoo’ (or total resting time for events without ‘rest hoo’ production) as the response variable and with the subject ID entered as a random factor. We ran GLMM4 on all resting bouts with the production of ‘rest hoo’ vocalisation by the subject (at least one ‘rest hoo’ produced/no ‘rest hoo’ produced) as the test variable. We ran GLMM5 on events with at least one ‘rest hoo’ produced by the subject and we used the immediate vocal response (at least one immediate vocal response/no vocal response) as the test variable.

We recorded some occurrences of the subject producing several ‘rest hoos’ during the same resting bout so we tested if the number of call produced affected the time the subject spent resting afterwards by analysing the resting bouts during which the subject produced at least one ‘rest hoo’. We ran a GLMM (GLMM6) with a gamma distribution and inverse link function, with the time spent resting (in minutes) after the subject produced a ‘rest hoo’ as the response variable and with the number of ‘rest hoo’ produced during the resting bout as the test variable. The subject ID, as well as the ID of the resting bout, were entered as random factors.

#### ‘Rest hoo’ perception

We then investigated what happened when the subjects heard a ‘rest hoo’ produced by another male.

We first explored the recipient’s behavioural response by testing if the subject rested longer after hearing another male producing a ‘rest hoo’. We analysed the events during which the subject did not vocalise and we conducted a GLMM (GLMM7) with a gamma distribution and inverse link function, with the time spent resting (in minutes) after the first ‘rest hoo’ was heard (or total resting time for events during which the subjects did not hear any ‘rest hoo’) as the response variable. The production of ‘rest hoo’ vocalisation by another male (at least one ‘rest hoo’ produced/no ‘rest hoo’ produced) was the test variable and the subject ID was entered as a random factor.

To investigate how the identity of the caller influenced the subject’s resting behaviour, we only further analysed events during which the subject heard ‘rest hoos’ from other males. We ran a GLMM (GLMM8) with a gamma distribution and inverse link function, with the time spent resting (in minutes) after the ‘rest hoo’ was produced as the response variable. Test variables were the number of individuals present, whether the caller was a high-ranking male (yes/no), one of the subject’s top three grooming partners (yes/no), or top three proximity partners (yes/no), as well as the interaction between the caller’s grooming index and his rank (Elo-rating). To control for the fact that several ‘rest hoos’ could be produced during the same resting bout, the remaining number of ‘rest hoo’ heard in the resting bout was the control variable and the subject ID, as well as the ID of the resting bout, were entered as random factors.

#### Model selection

For the GLM (GLM1) and two GLMMs (GLMM2 and 8), we used an exploratory approach, using several test variables. To disentangle the effect of each variable and determine which models fitted the data best, we used a statistical model selection approach. We first fitted several models with the test variables as fixed effects, then we selected the best model based on the lowest AICc (i.e., Akaike information criterion corrected; Burnham et al. 2011), using the *dredge* function of the ‘MuMIn’ R package version 1.43.17 (Barton 2020). Variables were considered to improve the fit of the model only if their removal from the model inflated the AICc value by more than two units (Burnham and Anderson 2004). Finally, we tested the significance of the selected models by comparing them to a corresponding null model (including control variables and, for the GLMMs, random factors), using likelihood ratio tests (LRT) (*lrtest* function of ‘lmtest’ package; Zeileis and Hothorn 2002). Only results of the selected models are presented in the results section below.

GLMMs were run using the *glmer* function of the ‘lme4’ R package version 1.1–21 (Bates et al. 2015). All analyses were implemented in R v3.6.1 (R Core Team 2019).

## Results

Across subjects (*N* = 10 adult males), we recorded *N* = 1494 resting bouts, *N* = 145 during which the subject produced at least one ‘rest hoo’ call (9.7%) and *N* = 147 during which another male produced at least one ‘rest hoo’ call (9.8%), over a study period of 141 days (690 h observation). We found that resting bouts lasted 10 min on average (range 15 s to 132 min). As mentioned, for all analyses involving social variables (i.e., rank, grooming index and preferred social partners) we only considered data collected before the outbreak, which consisted of *N* = 967 resting bouts, *N* = 101 during which the subject produced at least one ‘rest hoo’ call (10.4%) and *N* = 97 during which another male produced at least one ‘rest hoo’ call (10.0%).

### ‘Rest hoo’ production

The results of the GLMM1 showed that the presence of an audience was a significant predictor of ‘rest hoo’ call production (GLMM1; *p* < 0.001; Online Resource 1, Fig. S2 and Table S2a). When alone, focal animals produced ‘rest hoos’ in only 9 of 334 resting bouts (2.7%), whereas, in the presence of an audience, they produced ‘rest hoo’ calls in 136 of 1066 resting bouts (12.8%).

**Fig. 1:**
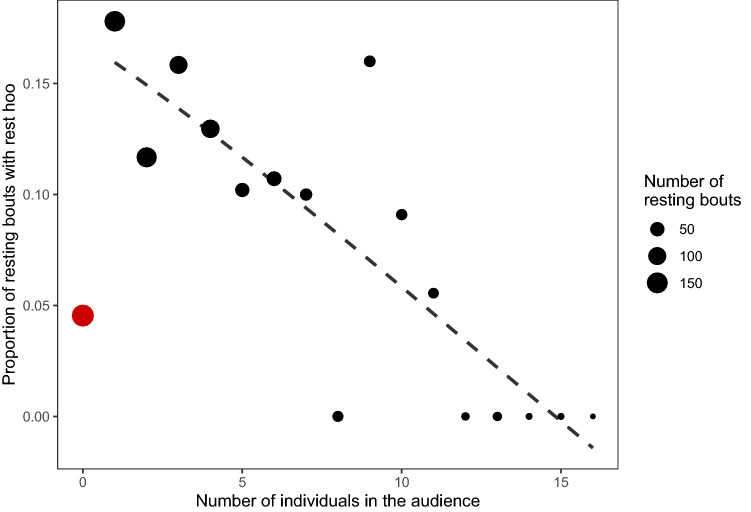
Relationship between the proportion of resting bouts with ‘rest hoo’ produced by the subject and the audience size (GLM1). The size of the points corresponds to the sample size (number of resting bouts recorded for each audience size). The red point represents the resting bouts when the subject was resting alone

**Fig. 2:**
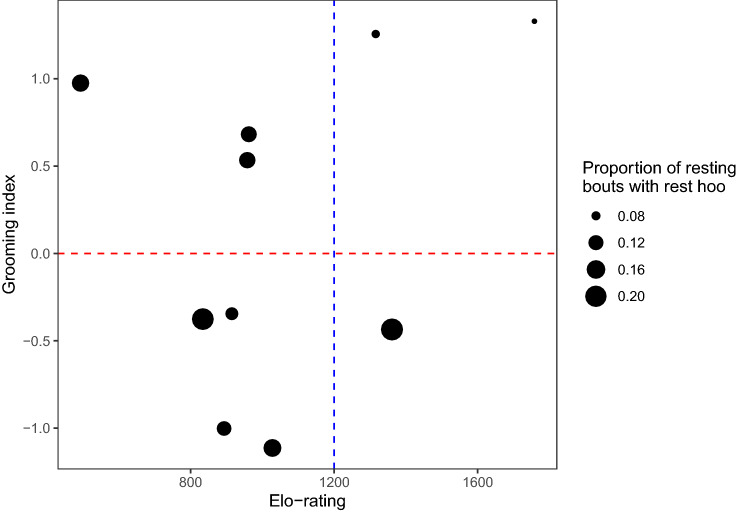
Subject’s grooming index and dominance rank (Elo-rating) and his propensity to produce ‘rest hoos’ during resting bouts, i.e., the number of resting bouts during which he produced a ‘rest hoo’ over the total number of resting bouts recorded for this subject (each point corresponds to one study subject). The dashed red line indicates the mean grooming index, with individuals above the line being higher groomers. The dashed blue line indicates the separation between the top three high-ranking males (on the right of the line) and the non-dominant ones. The highest proportions of resting bouts with ‘rest hoos’ produced are from either non-dominant males or ones with a below-average grooming index (GLM1)

When further investigating data collected before the outbreak, the results of the GLM1 (Online Resource 1, Table S2b) revealed that ‘rest hoo’ production could be predicted, significantly, by audience size, subjects calling more often with small rather than large audiences (*p* = 0.018; Fig. 1). It also showed that the interaction between the subject’s dominance rank and grooming index was a significant predictor of ‘rest hoo’ production, with high-ranking males with a high grooming index calling little and other males calling much (*p* = 0.022; Fig. 2).

**Fig. 3:**
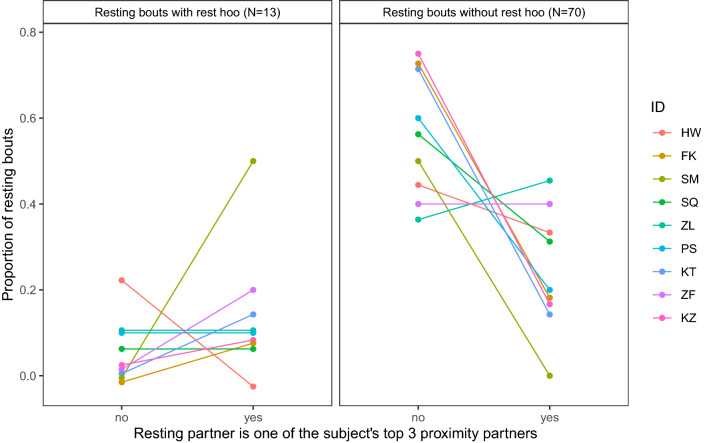
Proportion of dyadic resting bouts during which the subject produced a ‘rest hoo’ (left panel) or not (right panel) depending on whether the resting partner was one of the subject's top proximity partner or not, over the total number of dyadic resting bouts (*N* = 83), for each study subject (GLMM2). The subjects are ordered by dominance rank (from top high-ranking to bottom low-ranking)

When investigating male dyadic resting bouts (*N* = 83), the results of the GLMM2 (Online Resource 1, Table S2c) showed that ‘rest hoo’ production could be significantly predicted by the presence of one of the subject’s top three proximity partners rather than another male (*p* = 0.046; Fig. 3). When further investigating these male dyadic resting bouts, the results of GLMM3 showed that the presence of the resting partner at the end of the resting bout could not be significantly predicted by the production of ‘rest hoo’ (*p* = 0.889; Online Resource 1, Table S2d). Indeed, whether the subject produced a ‘rest hoo’ or not, his resting partner was present at the end of the resting bout in the majority of cases (approximately 80% of the resting bouts). One of the subject (MS) was not included in these analyses (GLMM2 and 3) since we did not have any data points with him resting alone with another male.

**Fig. 4:**
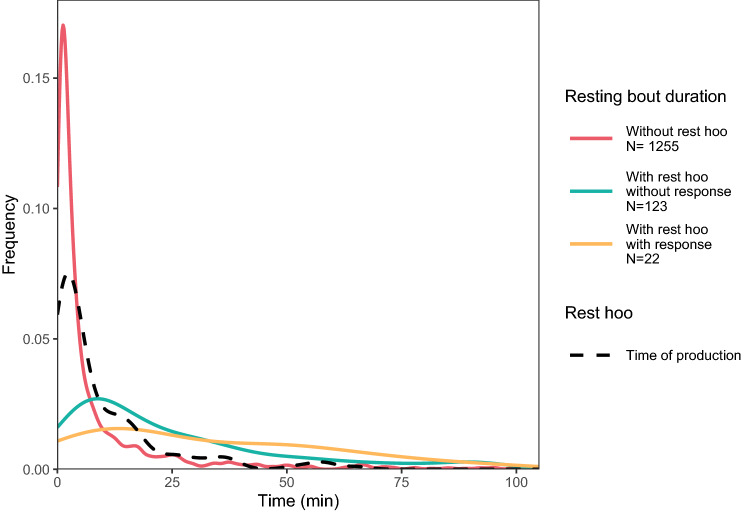
Distribution (kernel density estimates) of the time of ‘rest hoo’ production and of the time the subject spent resting depending on whether he produced a ‘rest hoo’ (GLMM4) and if this call elicited an immediate response by another individual or not (GLMM5)

Subjects produced a ‘rest hoo’ after resting 9.6 min on average, which was similar to the mean duration of silent resting bouts (i.e., when no call is produced) (8.0 min; Fig. 4). Moreover, focal subjects rested significantly longer after producing a ‘rest hoo’ than without one (GLMM4; *p* < 0.001; Fig. 4; Online Resource 1, Table S2e). Resting lasted longer if another male responded to the focal subject’s ‘rest hoo’ (GLMM5; *p* < 0.001; Fig. 4; Online Resource 1, Table S2f).

**Fig. 5:**
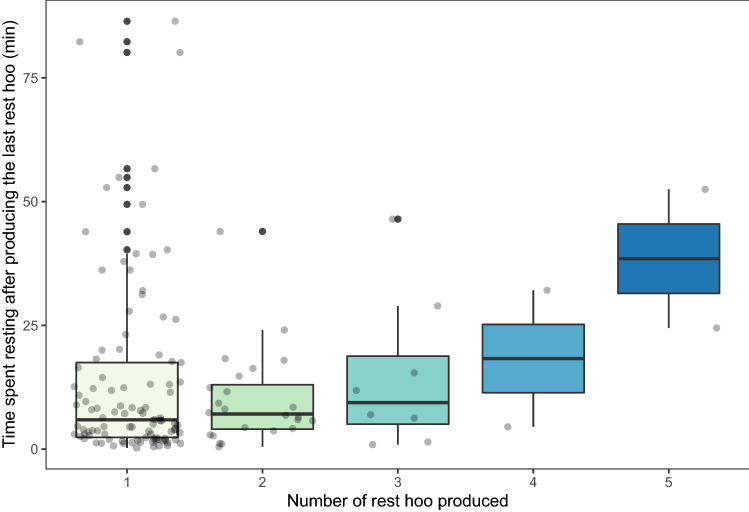
Time spent resting by the subject after he produced the last ‘rest hoo’ of the resting bout, depending on the total number of ‘rest hoo’ he produced during this resting bout (GLMM6)

During some resting bouts (*N* = 36), the subject produced more than one ‘rest hoo’, with up to five calls produced during the same resting bout. The results of the GLMM6 showed that the time the subject spent resting after producing a ‘rest hoo’ significantly increased with the number of ‘rest hoo’ produced (*p* < 0.001; Fig. 5; Online Resource 1, Table S2g).

### ‘Rest hoo’ perception

**Fig. 6:**
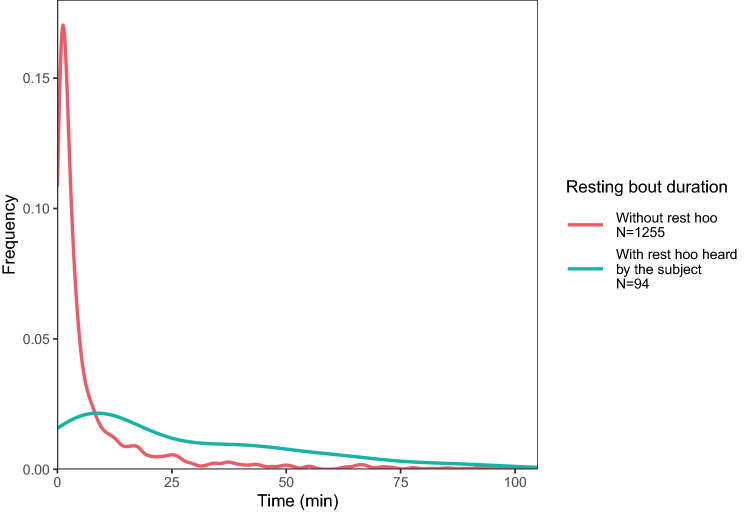
Distribution (kernel density estimates) of the time the subject spent resting depending on whether the subject heard a ‘rest hoo’ produced by another adult male or not (GLMM7). Only resting bouts during which the subjects did not produce a ‘rest hoo’ himself are displayed

**Fig. 7:**
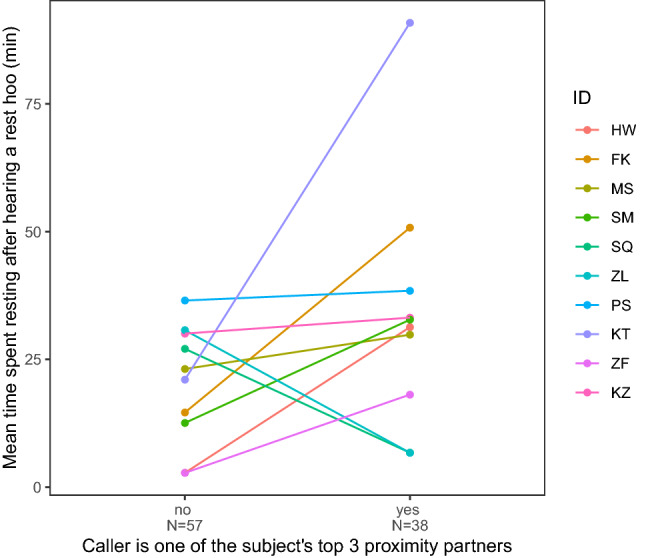
Mean time the subject spent resting after hearing a ‘rest hoo’ depending on whether the caller was one of the subject’s top three proximity partners or not (GLMM8). The subjects are ordered by dominance rank (from top high-ranking to bottom low-ranking)

The results of the GLMM7 showed that, when subjects heard a ‘rest hoo’ from another male, the subjects spent significantly more time resting afterwards than if no calls were produced or heard (GLMM7; *p* < 0.001; Fig. 6; Online Resource 1, Table S2h). The results of the GLMM8 showed that subjects also rested longer if the call was produced by one of the subject’s top three proximity partners rather than another male (*p* = 0.026; Fig. 7; Online Resource 1, Table S2i).

Finally, even though most ‘rest hoos’ were produced when both the caller and the recipients were resting (85.6%; 352 of 411 calls), on several occasions they were produced immediately (i.e., within the next 5 s) after other individuals were trying to leave (9.0%; *N* = 37) or in response to other group members passing by (5.4%; *N* = 22). Here, calling had its desired effect in a considerable number of cases (40.7%; *N* = 24), insofar as targeted individuals stopped travelling to join the resting caller (Online Resource 1, Table S3).

## Discussion

In this study, we were interested in exploring the context of production of ‘rest hoos’ by wild chimpanzee males of Budongo Forest and whether these calls had an intentional quality, i.e., whether they were emitted communicatively and with a particular goal. We found that ‘rest hoos’ were mainly produced when resting in small parties and towards the end of typical silent resting bouts, which have an average duration of about 8 min (Figs. 1, 4). Calls were mainly produced if subjects were with others and were significantly predicted by whether the audience contained one of the subjects’ top three proximity partners (Figs. 1, 3). Also, resting bouts with ‘rest hoos’ were significantly longer than silent bouts, and resting time increased with the number of calls produced (Figs. 4, 5, 6). Several social factors were also associated with prolonged resting bouts, notably if calls were answered by other group members (Fig. 2) and if the caller was one of the subject’s top three proximity partners (Fig. 7). Finally, although all ten males produced ‘rest hoos’, the behaviour was less common in high-ranking males with a high grooming index than in other males (Fig. 2). In the following sections, we discuss these results and develop our arguments supporting the claim that ‘rest hoos’ are produced intentionally to prolong resting with desirable partners, i.e., in a goal-directed way and in the presence of a specific audience. We also speculate that this call has probably evolved as an alternative strategy to tactile-based social bonding for less popular partners (i.e., non-dominant males with a low-grooming index).

Overall, we found that the large majority of resting bouts (almost 90%) were free of ‘rest hoos’, suggesting that the default way of making decisions about the timing of transition from resting to another activity is by other means. Chimpanzees may use other signals, not considered in this study, to influence spatial proximity and, thus, social cohesion. Gestural, facial, and multimodal signals may also play a role during resting bouts and there may be important inter-individual differences in how such signals are deployed, perhaps similar to how human infants assemble different signals into sequences during their illocutionary acts. Detailed observation of all communication channels, including follow-up behaviour, such as audience checking, would help to get a more comprehensive picture of how chimpanzees manage social distance.

Although our study focused on adult males of the Sonso community the same call is also produced by females and in other chimpanzee communities where researchers have paid specific attention (e.g., Gombe National Park, Tanzania: Goodall 1986). Therefore, future studies should investigate call production by other individuals and in other field sites, ideally using playback experiments to further investigate the behavioural responses to ‘rest hoos’.

Finally, our findings also showed that social relationships can be meaningfully described in terms of spatial proximity and grooming interactions, rather than computing a generalised ‘composite social index’ (Silk et al. 2013). Indeed, we found that subjects called more often when in the presence of a top proximity partner and rested longer after hearing a ‘rest hoo’ produced by a top proximity partner, whereas top grooming partners did not seem to affect either call production or behavioural responses. As we explained above, grooming and vocalising might actually be part of alternative individual social bonding strategies and, in line with other studies (Bray and Gilby 2020; Mitani 2009; Samuni et al. 2018, 2021), we suggest that proximity and grooming should be considered separately when investigating social bonds, as they may reflect qualitative differences in relationships.

### Intentional communication

Although authors differ in some key aspects, all definitions of intentional animal communication require some evidence of goal-directedness and of the signal being produced in the presence of an audience (Graham et al. 2019; Leavens et al. 2005; Townsend et al. 2017; Zuberbühler and Gomez 2018). In our study, ‘rest hoos’ were almost always produced in the presence of an audience (Fig. 1), which suggests that chimpanzees have voluntary control over the production of ‘rest hoos’, as it has also been argued for the two other ‘hoo’ variants (‘travel hoos’: Gruber and Zuberbühler 2013; ‘alert hoos’: Schel et al. 2013b) and other chimpanzee vocalisations (Hopkins et al. 2007; Leavens et al. 2004; Schel et al. 2013a). Moreover, ‘rest hoos’ seemed to be preferentially produced in the presence of one of the subject’s top three proximity partners (Fig. 3). These evidence all seem to suggest that ‘rest hoos’ were directed towards an audience, and even towards specific individuals (i.e., top proximity partners). More crucially, similar to intentional vocalisations in human infants, chimpanzee ‘rest hoos’ were not produced to refer to external stimuli, but data suggest they were deployed as a way to convey the caller’s apparent desire to prolong an already ongoing resting. However, we also found that in about 15% of cases, calls appeared to be given to keep others from leaving or to persuade bypassing individuals to join the resting party (which was often successful; Online Resource 1, Table S3). Our results show that subjects rested for longer after calling than not calling (Fig. 4). Also, we suggest that ‘rest hoo’ production is part of a persuasion effort to influence recipients as, in a considerable number of cases, focal animals called multiple times during the same resting bout which was again associated with longer resting periods (Fig. 5). This interpretation is further supported by data showing that individual calls were sometimes immediately answered, perhaps expressions of agreement and thus shared intention, which explains why answered calls were associated with prolonged resting bouts (Fig. 4). Such speculations could provide interesting avenues for future research. Finally, recipients rested longer after hearing a ‘rest hoo’ produced by another male (Fig. 6), suggesting that the call elicited a behavioural response in line with the signaller’s goal, i.e., to prolong resting. These findings provide strong evidence that ‘rest hoos’ are produced with a specific goal, i.e., to prolong an ongoing period of resting with particular male partners.

One further prediction of the intentionality hypothesis is that, following ‘rest hoos’, callers should be more likely to terminate resting bouts together (since callers appear to address particular recipients in the audience). However, we found that, when resting with one other male, subjects usually did not keep resting alone after their resting partners departed (only 20% of resting bouts), whether they produced a ‘rest hoo’ or not (GLMM3). We think this result shows that males often depart together and thus try to keep cohesion with their social partners. If males indeed produce ‘rest hoos’ to prolong resting with their partners then it would make sense that ‘rest hoo’ production would affect the duration of resting bouts but not necessarily the presence of the partner at the end of the resting bout. Indeed, subjects might want to keep cohesion with their partners even when they do not specifically wish to rest (hence, no use for ‘rest hoo’ vocalisations) and would thus depart and travel with them. However, our study did not consider departing patterns (i.e., which individual stopped resting first and started travelling) and we think this should be explored further. Alternatively, it is conceivable that males only produced ‘rest hoos’ when cohesion was threatened, again resulting in joint resting with or without calls. We also think that, similarly to previous studies that investigated cohesion when travelling or feeding (Gruber and Zuberbühler 2013; Schel et al. 2013a), our study focused on one activity, i.e., resting, and future research should investigate overall cohesion between males and focus on association patterns across activities.

### Evolution and function

In non-human primates, increases in vocal repertoire sizes are correlated with both increases in group size and time spent grooming (McComb and Semple 2005). Dunbar (1998) argued that, with increasing group size, the time required to maintain relations via grooming would reach a threshold due to time constraints (Lehmann et al. 2007). Amongst non-human primates, chimpanzees live in large groups, suggesting that this species has been under selection pressure to evolve other ways to maintain social cohesion. Recent studies in other primate species support this view and show that contact calls can be produced preferentially towards social partners, equivalent to “grooming-at-a-distance” (Japanese macaques: Arlet et al. 2015; lemurs: Kulahci et al. 2015; spider monkeys: Briseno-Jaramillo et al. 2018). Our data are in line with these findings since subjects produced ‘rest hoos’ preferentially in the presence of one of their top three proximity partners (Fig. 3) and rested longer after hearing ‘rest hoos’ produced by such partners (Fig. 7). Even more relevant here is the fact that some individuals appear to prefer the vocal over the tactile route since callers were mainly low groomers. Whether this was due to individual differences or differences in the social position will have to be investigated with further research. Lower ranking males might also need to make more effort to maintain cohesion with other males, compared to higher-ranking ones, which would explain why they would produce more ‘rest hoos’. Indeed, maintaining cohesion with higher-ranking individuals might be beneficial to form coalitions, or to have access to mates (Bray et al. 2016; Muller and Mitani 2005). This call could, therefore, be an alternative strategy deployed by less popular partners (i.e., all but high grooming and high-ranking individuals) as a substitute for other forms of social desirability leading to social cohesion.

## Conclusions

Our results plausibly suggest that ‘rest hoos’ are produced intentionally to prolong resting and promote social cohesion, particularly with top proximity partners. These vocalisations might be an alternative strategy to tactile-based social bonding and, thus, bring more evidence that social cohesion and bonding through vocal communication has emerged in highly social species with large groups, due to group size and time constraints. These results bring to light an interesting relationship and interaction between tactile and vocal behaviours and suggest that future research on communication should adopt a multimodal approach (Fröhlich and van Schaik 2018; Liebal et al. 2013; Slocombe et al. 2011).

This study is currently the only in-depth study investigating the function of ‘rest hoo’ vocalisations, exploring the conditions in which it is produced by adult males and how this call affects the duration of resting bouts. Our results help to better understand the communication system of our closest relatives and provide interesting avenues for future research.

## Supplementary Information

Below is the link to the electronic supplementary material.

Supplementary file1 (DOCX 288 KB)

Supplementary file2 (WAV 147 KB)

Supplementary file3 (WAV 395 KB)

## Data Availability

The data supporting this article can be found in the following Figshare repository: https://figshare.com/projects/An_intentional_cohesion_call_in_male_chimpanzees_of_Budongo_Forest/97508.

## Code availability

The code used for this study is available from the corresponding author on reasonable request.
