# Supplementary material for: An intentional cohesion call in male chimpanzees of Budongo Forest
Source: Anim Cogn. 2022 Jan 19;25(4):853–66. doi: 10.1007/s10071-022-01597-6 (PMC9334450; doi:10.1007/s10071-022-01597-6)
Supplement: Supplementary file 1 — Supplementary file1 (DOCX 288 KB) [file 10071_2022_1597_MOESM1_ESM.docx]

**Electronic Supplementary Material 1**

**Title:** An intentional cohesion call in male chimpanzees of Budongo Forest.

**Journal:** Animal Cognition

**Authors:** Alice Bouchard & Klaus Zuberbühler

**Corresponding author:** Alice Bouchard (alicebouchard6@gmail.com)

**Affiliation:** Institute of Biology, University of Neuchatel, Neuchatel, Switzerland; Budongo Conservation Field Station, Masindi, Uganda


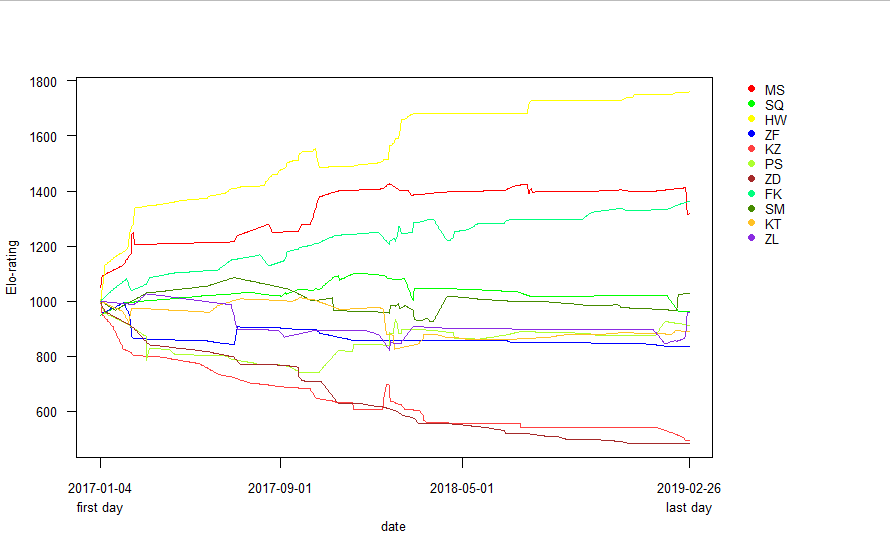
 **Fig. S1** Evolution of the Elo-rating scores of the 11 adult males of the Sonso community from 12 months before the start of the study (January 4^th^ 2017) to the end of the first study period (February 26^th^ 2019). In contrast to traditional matrix-based assessments, elo-rating conceptualises rank as a dynamic variable that changes with each social interaction. Each male starts the process with a fixed score, which is continuously updated following each dyadic interaction. In particular, each time a male produces pant grunts to another male, he loses points whereas the recipient gains the same number of points. The number of points gained or lost depends on the expected outcome, which is calculated prior the interaction. Unexpected outcomes lead to more point changes than expected outcomes (Elo 1978; Neumann et al. 2011).

**Table S1** Top 3 grooming partners and top 3 proximity partners, with the associated DSI_P_ and DSI_G_ values respectively, for each of the study subjects. The DSI_P_ and DSI_G_ calculations were derived from the DSI introduced by Silk et al. (2013) which attributes a value of 1 to the average social bond across all dyads in the group (in our case 55 dyads; N=11 males). If a dyad has a value superior (or inferior) to 1, the dyad is considered to have a stronger (or weaker) social bond than average. However, in this study, we did not use the value of the group mean (1) to compare DSI, but we established each subject’s preferred partners by selecting the three highest DSI values (both for DSI_G_ and DSI_P_) for each subject. We chose this method to account for the fact that some individuals are intrinsically more social than others, i.e., a very social male will have most of its DSIs above 1 whereas an asocial male will have most of his DSIs below one.

| **Focal ID** | **Top 3 grooming partners** | | | | | | **Top 3 proximity partners** | | | | | |
| --- | --- | --- | --- | --- | --- | --- | --- | --- | --- | --- | --- | --- |
|  | *ID1* | *DSI_G_* | *ID2* | *DSI_G_* | *ID3* | *DSI_G_* | *ID1* | *DSI_P_* | *ID2* | *DSI_P_* | *ID3* | *DSI_P_* |
| FK | HW | 2.506 | MS | 0.82049 | ZL | 0.72276 | HW | 1.93591 | PS | 1.23312 | SM | 1.11567 |
| HW | MS | 7.41631 | ZL | 3.56719 | SQ | 2.86252 | KT | 3.14544 | MS | 2.96374 | ZL | 2.74109 |
| KT | HW | 2.10979 | MS | 1.8699 | ZL | 0.91899 | HW | 3.14544 | MS | 1.54579 | FK | 1.08609 |
| KZ | ZL | 1.18471 | PS | 0.87028 | SM | 0.80654 | SQ | 1.38666 | PS | 0.94672 | ZF | 0.90951 |
| MS | HW | 7.41631 | PS | 2.71263 | SQ | 2.05929 | HW | 2.96374 | SQ | 1.97565 | KT | 1.54579 |
| PS | MS | 2.71263 | HW | 2.36197 | ZL | 1.8472 | HW | 1.57758 | FK | 1.23312 | MS | 1.1528 |
| SM | HW | 1.58801 | ZL | 1.47764 | PS | 0.80995 | HW | 1.93149 | ZD | 1.26756 | ZL | 1.15455 |
| SQ | HW | 2.86252 | MS | 2.05929 | PS | 1.3291 | MS | 1.97565 | HW | 1.78858 | KZ | 1.38666 |
| ZD | ZL | 2.14204 | PS | 1.34719 | SQ | 1.1774 | ZL | 1.4993 | SM | 1.26756 | SQ | 1.01107 |
| ZF | MS | 0.82979 | HW | 0.66267 | ZL | 0.4642 | HW | 1.78382 | SQ | 0.98214 | KZ | 0.90951 |
| ZL | HW | 3.56719 | ZD | 2.14204 | PS | 1.8472 | HW | 2.74109 | ZD | 1.4993 | SM | 1.15455 |

**Table S2** Coefficients and significance of the variables entered in

1. the GLMM with a Binomial distribution to analyse whether the presence of an audience affected ‘rest hoo’ production (GLMM1).
2. the GLM with a Binomial distribution to analyse which parameters influenced hoo production by the focal adult males during resting bouts (GLM1). A model selection approach was used with this model.
3. the GLMM with a Binomial distribution to analyse which social parameters influenced hoo production by the focal adult males during male dyadic resting bouts (GLMM2). A model selection approach was used with this model.
4. the GLMM with a Binomial distribution to analyse whether the production of ‘rest hoo’ by the subject affected the presence of the resting partner at the end of male dyadic resting bouts (GLMM3).
5. the GLMM with a Gamma distribution to analyse whether the production of ‘rest hoo’ by the subject affected the time he spent resting (GLMM4).
6. the GLMM with a Gamma distribution to analyse whether immediate vocal responses to ‘rest hoo’ produced by the subject affected the time he spent resting (GLMM5).
7. the GLMM with a Gamma distribution to analyse whether the number of ‘rest hoo’ produced by the subject affected the time he spent resting (GLMM6).
8. the GLMM with a Gamma distribution to analyse whether the reception of ‘rest hoo’ (i.e., produced by other individuals) affected the time the subject spent resting (GLMM7).
9. the GLMM with a Gamma distribution to analyse which parameters influence the time the focal individual spent resting after hearing a rest hoo vocalisation produced by another adult male (GLMM8). A model selection approach was used with this model.

| *(a) GLMM 1 - Probability of the focal individual calling depending on the presence of an audience* | | | | |
| --- | --- | --- | --- | --- |
| Variables | Estimate | SE | Z | *p* |
| Presence of an audience (Yes) | 1.67 | 0.35 | 4.76 | <0.001 |
| Random effect: Subject ID (Variance=0.01, SD=0.10) | | | | |
|  |  |  |  |  |
| (b) *GLM1 - Probability of the focal individual calling* (Best fitting model*: AICc = 487) | | |  |  |
| Variables | Estimate | SE | Z | *p* |
| Duration of the resting event | 0.04 | 0.01 | 7.70 | <0.001 |
| Number of individuals in the audience | -0.11 | 0.05 | -2.37 | 0.018 |
| Grooming index | -0.24 | 0.15 | -1.60 | 0.110 |
| Dominance rank (Elo-rating) | 0.28 | 0.21 | 1.33 | 0.183 |
| Grooming index * Dominance rank | -0.46 | 0.20 | -2.28 | 0.022 |
| Full model: Duration of the resting event + number of individuals in the audience + presence of a female (yes / no) + presence of a high-ranking male (yes / no) + presence of a top 3 grooming partner (yes / no) + presence of a top 3 proximity partner (yes / no) + grooming index * dominance rank | | | | |
| * χ² tests for the log-likelihood ratios, best fitting model–null model, χ² = 15.69, *p* = 0.003 | | | | |
|  |  |  |  |  |
| (c) *GLMM 2 - Probability of the focal individual calling when resting in male dyads* (Best fitting model*: AICc = 74) | | | | |
| Variables | Estimate | SE | Z | *p* |
| Resting partner is a top 3 proximity partner (Yes) | 1.25 | 0.63 | 2.00 | 0.046 |
| Full model: Duration of the resting event + resting partner is a high-ranking male (yes / no) + resting partner is a top 3 grooming partner (yes / no) + resting partner is a top 3 proximity partner (yes / no) | | | | |
| * χ² tests for the log-likelihood ratios, best fitting model–null model, χ² = 0.12, *p* < 0.001; Random effect: Subject ID (Variance=0.00, SD=0.00) | | | | |
|  |  |  |  |  |
| (d) GLMM3 - *Presence of the resting partner at the end of male dyadic resting bouts* | | | | |
| Variables | Estimate | SE | Z | *p* |
| Production of 'rest hoo' (Yes) | -0.12 | 0.85 | -0.14 | 0.889 |
| Resting time after producing a 'rest hoo' | -0.03 | 0.02 | -2.10 | 0.035 |
|  |  |  |  |  |
| *(e) GLMM 4 - Resting time depending on 'rest hoo' production* | | | | |
| Variables | Estimate | SE | Z | *p* |
| Production of 'rest hoo' (Yes) | -0.07 | 0.01 | -9.39 | <0.001 |
| Random effect: Subject ID (Variance=0.00, SD=0.02) | | | | |
|  |  |  |  |  |
| *(f) GLMM 5 - Resting time depending on immediate vocal response* | | | | |
| Variables | Estimate | SE | Z | *p* |
| Immediate vocal response (Yes) | -0.017 | 0.003 | -6.53 | <0.001 |
| Random effect: Subject ID (Variance=0.00, SD=0.02) | | | | |
|  |  |  |  |  |
| *(g) GLMM 6 - Resting time depending on the number of 'rest hoo' produced* | | | | |
| Variables | Estimate | SE | Z | *p* |
| Number of 'rest hoo' produced | 0.006 | 0.002 | 3.59 | <0.001 |
| Random effects: Subject ID (Variance=0.00, SD=0.00) and Resting bout ID (Variance=0.01, SD=0.10) | | | | |
|  |  |  |  |  |
| *(h) GLMM 7 - Resting time depending on 'rest hoo' reception* | | | | |
| Variables | Estimate | SE | Z | *p* |
| Immediate vocal response (Yes) | -0.075 | 0.008 | -9.64 | <0.001 |
| Random effect: Subject ID (Variance=0.00, SD=0.00) | | | | |
|  |  |  |  |  |
| (i) GLMM8 - *Resting time after hearing a rest hoo* (Best fitting model*: AICc = 800) | | | | |
| Variables | Estimate | SE | Z | *p* |
| Remaining number of hoos in the resting bout | -0.007 | 0.002 | -4.38 | <0.001 |
| Caller is a top 3 proximity partner (yes) | -0.019 | 0.008 | -2.23 | 0.026 |
| Full model: remaining number of hoos in the resting bout + number of individuals present + caller is a high-ranking male (yes / no) + caller is a top 3 grooming partner (yes / no) + caller is a top 3 proximity partner (yes / no) + caller’s sociability * caller's rank | | | | |
| * χ² tests for the log-likelihood ratios, best fitting model–null model, χ² = 4.42, *p* = 0.035; Random effects: Subject ID (Variance=0.00, SD=0.00) and Resting bout ID (Variance=0.00, SD=0.00) | | | | |


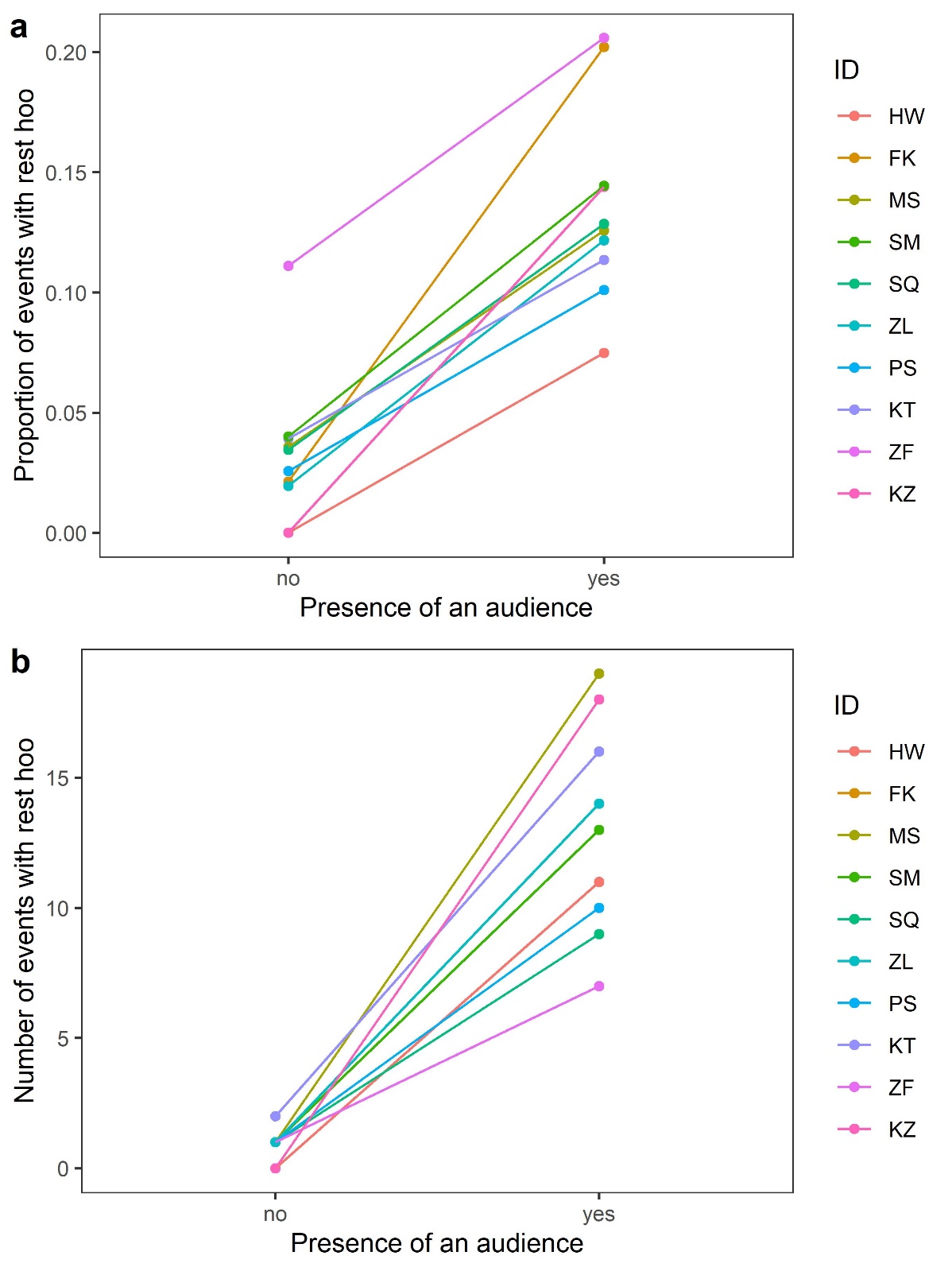


**Fig. S2 a** Proportion and **b** raw numbers of events with at least one rest hoo produced by the focal individual depending on the presence of a potential audience for each study subject (GLMM1). The subjects are ordered by dominance rank (from top high-ranking to bottom low-ranking).

**Table S3** Behaviour of the recipients immediately (i.e., within 5s) before and after the subject produced a ‘rest hoo’. We recorded the number of events when there was no change in the activity of the audience (i.e., everyone was resting), when at least one individual in the audience started traveling or when individuals were passing by, before the subject produced a ‘rest hoo’. For the last two categories, we also recorded whether the traveling individual kept traveling or joined the subject resting after he produced the ‘rest hoo’.

| Receiver's behaviour **before** call production | Receiver's behaviour **after** call production | Number of events | |
| --- | --- | --- | --- |
| Rests | | 352 | |
| Initiates travel | starts resting | 10 | 37 |
|  | keeps traveling | 27 |  |
| Travels | starts resting | 14 | 22 |
|  | keeps traveling | 8 |  |
| TOTAL | | 411 | |

**References**

Elo AE (1978) The rating of chess players, past and present. Arco Pub., New York, NY

Neumann C, Duboscq J, Dubuc C, et al (2011) Assessing dominance hierarchies: validation and advantages of progressive evaluation with Elo-rating. Anim Behav 82:911–921. https://doi.org/10.1016/j.anbehav.2011.07.016

Silk J, Cheney D, Seyfarth RM (2013) A practical guide to the study of social relationships. Evol Anthropol Issues, News, Rev 22:213–225. https://doi.org/10.1002/evan.21367
